# Comprehensive assessment of the genotype-phenotype spectrum and treatment for monoallelic insulin receptoropathy in a cohort of 505 people

**DOI:** 10.21203/rs.3.rs-10884175/v1

**Published:** 2026-09-28

**Authors:** Rebecca J Brown, Jingxiang Xu, Jian’an Luan, Bruno Donadille, Vahid Aslanzadeh, Christine Bellanné-Chantelot, Valeria Grasso, Delphine Collin-Chavagnac, Mafalda Mucciolo, Ingrid Allix, Anne Bachelot, Sabine Baron, Danila Benevento, Laurence Blanco, Muriel Bourgeon, Sophie Christin-Maitre, Elaine Cochran, Régis Coutant, Lea Dehghani, Annalisa Deodati, Charlotte Djakouré, Danièle Dubois-Laforgue, Lise Duranteau, Claire-Lise Gay, Siri Atma W Greeley, Tulay Guran, Balamurugan Kandasamy, Marie Laloi-Michelin, Pascale Mahot, Dominique Maiter, Viswanathan Mohan, Rinki Murphy, Estelle Nobécourt, Novella Rapini, Sylvie Salenave, Pratik Shah, Megan Startzell, Igor Tauveron, Daniel Terral, A. Kemal Topaloglu, Praveen V.P., Marie-Christine Vantyghem, Venkatesan Radha, Martin Wabitsch, Peter Winocour, Hannele Yki-Järvinen, Jacques Young, Rachel Williams, Kashyap Patel, Loredana Marcovecchio, Louis Lebreton, Camille Vatier, Nicholas Wareham, Anna Stears, Elise Bismuth, David Savage, Isabelle Jéru, Stephen O’Rahilly, Kevin Colclough, Gemma V. Brierley, Fabrizio Barbetti, Corinne Vigouroux, Robert K Semple

**Affiliations:** 1 Diabetes, Endocrinology and Obesity Branch, National Institute of Diabetes and Digestive and Kidney Diseases, National Institutes of Health, Bethesda, Maryland, USA;; 2 Institute for Genetics and Cancer; Institute for Neuroscience and Cardiovascular Research, University of Edinburgh, Edinburgh, UK;; 3 Institute for Neuroscience and Cardiovascular Research; Institute for Genetics and Cancer, University of Edinburgh, Edinburgh, UK;; 4 IMS Epidemiology, Institute of Metabolic Science, University of Cambridge, Cambridge, UK;; 5 Assistance Publique-Hôpitaux de Paris, Saint-Antoine Hospital, National Reference Center for Rare Diseases of Insulin Secretion and Insulin Sensitivity (PRISIS), Endocrinology, Diabetology and Reproductive Endocrinology Department, Sorbonne University, European Reference Network for Rare Endocrine Conditions (Endo-ERN Assistance-Publique Consortium), Paris, France;; 6 Sorbonne University, Inserm UMR_S938, Saint-Antoine Research Centre, Institute of Cardiometabolism and Nutrition, Paris, France;; 7 European Reference Network for Rare Endocrine Conditions (Endo-ERN Assistance-Publique Consortium), Paris, France;; 8 Assistance Publique-Hôpitaux de Paris, Pitié-Salpêtrière Hospital, Medical Genetics Department, Sorbonne University, Paris, France;; 9 Department of Experimental Medicine, University of Rome Tor Vergata, Rome, Italy; Bambino Gesù Children's Hospital, IRCCS, Rome, Italy;; 10 Hospices Civils de Lyon HCL, Biochemistry and Molecular Biology Department, CHU Lyon, Lyon, France;; 11 CarMeN Laboratory, Claude Bernard Lyon 1 University, Inserm, INRAE, Pierre-Bénite, France;; 12 Translational Cytogenomics Research Unit, Laboratory of Medical Genetics, Bambino Gesù Children's Hospital, IRCCS, Rome, Italy;; 13 Angers University Hospital, Endocrinology Diabetology and Nutrition Department, Angers, France;; 14 Assistance Publique-Hôpitaux de Paris, Pitié-Salpêtrière Hospital, Endocrinology and Reproductive Medicine Department, IE3M, European Reference Network for Rare Endocrine Conditions (Endo-ERN Assistance-Publique Consortium), Paris, France;; 15 Sorbonne University, Inserm CIC 2503 Pitié Salpêtrière, Paris, France;; 16 Nantes University, University Hospital, Paediatric Endocrinology Department, Nantes, France;; 17 Monogenic Diabetes Clinic, Endocrinology and Diabetes Unit, Bambino Gesù Children's Hospital, IRCCS, Rome, Italy;; 18 Department of Systems Medicine, University of Rome Tor Vergata, Rome, Italy;; 19 Bordeaux University Hospital, Endocrinology Diabetology Nutrition Department, Bordeaux, France;; 20 Assistance Publique-Hôpitaux de Paris, Antoine Béclère Hospital, Diabetology Unit, Internal Medicine and Immunology Department, Paris-Saclay University, Clamart, France;; 21 Assistance Publique-Hôpitaux de Paris, Saint-Antoine Hospital, Endocrinology, Diabetology and Reproductive Endocrinology Department, European Reference Network for Rare Endocrine Conditions (Endo-ERN Assistance-Publique Consortium), Paris, France;; 22 Sorbonne University, European Reference Network for Rare Endocrine Conditions (Endo-ERN Assistance-Publique Consortium), Inserm UMR_S933, Paris, France;; 23 Angers University Hospital, Pediatric Endocrinology Department, Angers, France;; 24 Assistance Publique-Hôpitaux de Paris, Cochin Hospital, Diabetology and Clinical Immunology Department, Paris-Cité University, Paris, France;; 25 La Rochelle Hospital, Endocrinology-Diabetology Unit, La Rochelle, France;; 26 INSERM U1016, Cochin Institute, Paris, France;; 27 Assistance Publique-Hôpitaux de Paris, Bicêtre Hospital, Medical Gynecology Department, Paris-Saclay University, Le Kremlin-Bicêtre, France;; 28 Hospices Civils de Lyon HCL, Pediatric Endocrinology Department, Bron, France;; 29 Kovler Diabetes Center, Section of Adult and Pediatric Endocrinology, Diabetes, and Metabolism, University of Chicago, Chicago, USA;; 30 Marmara University, School of Medicine, Department of Pediatric Endocrinology and Diabetes, Istanbul, Turkey;; 31 Assistance Publique-Hôpitaux de Paris, Lariboisière Hospital, Diabetology and Endocrinology Department, Paris, France;; 32 Nantes University Hospital, Endocrinology Diabetology Nutrition Department, Nantes, France;; 33 University Hospital Saint-Luc, UC Louvain, Endocrinology and Nutrition Department, European Reference Network for Rare Endocrine Conditions, Brussels, Belgium;; 34 Dr. Mohan's Diabetes Specialities Centre (IDF Centre of Excellence in Diabetes Care); Madras Diabetes Research Foundation, (ICMR Collaborating Centre of Excellence), Chennai, India;; 35 Department of Medicine, Faculty of Medical and Health Sciences, University of Auckland, Private Bag 92019, Auckland 1142, New Zealand;; 36 La Reunion University, University Hospital, Endocrinology, Diabetes and Nutrition Department, GHSR, Saint-Pierre de La Réunion, France;; 37 Inserm UMR 1188 Diabète Athérothrombose Thérapies Réunion Océan Indien (DéTROI), Saint-Pierre de La Réunion, France;; 38 Assistance Publique-Hôpitaux de Paris, Bicêtre Hospital, Reproductive Endocrinology Department, Le Kremlin-Bicêtre, France;; 39 Paris-Saclay University, Inserm UMR-U1185, Le Kremlin-Bicêtre, France;; 40 The Royal London Children's Hospital, Barts Health NHS Trust; Queen Mary University of London, London, UK;; 41 Clermont-Ferrand University Hospital, Endocrinology Department, Clermont Auvergne University, Clermont-Ferrand, France;; 42 Clermont-Ferrand University Hospital, Paediatric Department, Clermont-Ferrand, France;; 43 Massachusetts General Hospital, Division of Pediatric Endocrinology, Harvard Medical School, Boston, MA, USA;; 44 Amrita Institute of Medical Sciences, Cochin, India;; 45 University Hospital of Lille, Diabetes and Nutrition Department, EGID Center, University of Lille, Lille, France;; 46 Madras Diabetes Research Foundation, Department of Molecular Genetics, Chennai, India;; 47 Division of Pediatric Endocrinology and Diabetes, Department of Pediatrics and Adolescent Medicine, University Medical Center Ulm, Ulm, Germany;; 48 East and North Hertfordshire NHS Trust, Queen Elizabeth II Hospital, Welwyn Garden City, UK;; 49 University of Helsinki, Department of Medicine (HUS Inpatient and Rehabilitation);; 50 Minerva Medical Research Institute, Helsinki, Finland;; 51 Nottingham Children's Hospital, Nottingham University Hospitals NHS Trust, Nottingham, UK;; 52 Institute of Biomedical and Clinical Science, University of Exeter, Exeter, UK;; 53 Department of Paediatrics; University of Cambridge, Cambridge, UK;; 54 National Severe Insulin Resistance Service, Cambridge University Hospitals NHS Foundation Trust, Cambridge, UK;; 55 Bordeaux University Hospital Pellegrin, Biochemistry Department, Bordeaux, France;; 56 European Reference Network for Rare Endocrine Conditions (Endo-ERN Assistance-Publique Consortium), Inserm UMR_S938, Saint-Antoine Research Centre;; 57 Inserm UMR_S938, Saint-Antoine Research Centre, Institute of Cardiometabolism and Nutrition, Paris, France;; 58 Assistance Publique-Hôpitaux de Paris, Robert Debré Hospital, Pediatric Endocrinology and Diabetes Department, Paris, France;; 59 Reference Center for Rare Diseases of Insulin Secretion and Insulin Sensitivity (PRISIS), Paris-Cité University, Paris, France;; 60 MRC Metabolic Diseases Unit, Institute of Metabolic Science, University of Cambridge, Cambridge, UK;; 61 Royal Devon University Healthcare Foundation Trust, Exeter, UK;; 62 Department of Comparative Biomedical Sciences, Royal Veterinary College, London, UK

**Keywords:** INSR, Insulin Receptor, Insulin Resistance, Type A Insulin Resistance, Hypoglycaemia, Diabetes, Genetics, Hyperinsulinism, Growth, Pregnancy

## Abstract

**Background:**

Insulin signalling is mediated by the *INSR*-encoded receptor tyrosine kinase. Biallelic *INSR* variants cause the most severe insulin resistance (IR), whereas heterozygous mutations cause less rare but still severe IR. Dominantly inherited IR has been attributed to dominant negative action of intracellular (IC) missense variants, but whether extracellular (EC) variants exert dominant-negative effects, and whether simple heterozygous loss of function (LoF) causes a metabolic phenotype, remains unresolved. We aimed to clarify the phenotypic spectrum, underlying mechanisms, and natural history of IR caused by heterozygous *INSR* variants.

**Methods:**

We aggregated genetic and clinical data from 505 individuals with monoallelic rare *INSR* variants from published reports, international referral centres, ClinVar/HGMD, and the Fenland population cohort. Loss-of-function (LoF) status was assigned using in silico tools including NMDetector and SpliceAI; Insulin concentrations were compared against age-, sex-, and BMI-specific reference centiles (Fenland, NHANES, IDEFICS). The common p.M1180K variant was functionally characterised by assessing cell-surface expression, and insulin-induced autophosphorylation.

**Results:**

240 probands and 265 relatives were studied. There was marked female predominance among probands (Male:Female = 1:3.4) and earlier female presentation, most often with hyperandrogenism. Presentation with diabetes, hyperinsulinaemia, or hypoglycaemia was common in both sexes. Missense variants were 14-fold enriched in the IC domain compared to gnomAD, concentrated in key functional motifs including activation and catalytic loops, whereas LoF variants were distributed in proportion to domain length. The p.M1180K variant was normally expressed but autophosphorylation-deficient. Most EC variant carriers — including genetically-ascertained individuals with EC LoF or deleterious missense variants — showed moderate to severe IR from puberty onwards. Across the whole cohort with IC or EC variants, diabetes occurred in 56% and hypoglycaemia in 55%, the latter a presenting feature in 11% of probands and common among relatives. Birthweight standard deviation score (SDS) was reduced (–1.0) and BMI SDS modestly increased (+0.7). Hyperandrogenism and/or Polyendocrine Metabolic Ovarian Syndrome affected 82% of postpubertal females, while GDM affected 20–53% of pregnancies. Lipid profiles were normal.

**Conclusions:**

The human *INSR* demonstrates haploinsufficiency, with EC LoF and deleterious missense variants causing IR with variable penetrance. Hypoglycaemia is more common than previously recognised, warranting caution with diabetes therapies.

## BACKGROUND

Insulin is the crucial, nutritionally-entrained orchestrator of the physiological responses to food ingestion. Its widespread metabolic and mitogenic effects are mediated by a homodimeric, membrane-associated receptor of the tyrosine kinase class, encoded by the *INSR* gene (1, 2). Upon binding insulin, allosteric remodelling of the receptor induces phosphorylation of tyrosine residues within the intracellular domains of both receptor monomers. This phosphorylation occurs in trans - that is, phosphorylation of each monomer is carried out by the partner monomer in the dimeric receptor (3–5).

Human pathogenic variants in *INSR* were described in individuals with severe insulin resistance (IR) in 1988 (6, 7), shortly after reporting of the sequence of the *INSR* gene (1, 2). In subsequent years the clinical range of *INSR*-related disorders, and the causal mutational spectrum, were quickly delineated (8). The rarest and most severe insulin receptoropathies are caused by biallelic *INSR* variants, and most commonly present neonatally or in early childhood (9). Severe IR caused by heterozygous *INSR* variants is considerably more common and likely under-diagnosed. Variants associated with dominantly inherited severe IR are currently understood almost always to lie in the intracellular (IC) domain (10), consistent with a dominant negative mechanism. The prevailing explanation invokes impaired function of both mutated receptor homodimers, and also of heterodimers containing one wildtype and one mutant half receptor (10–14). In a heterozygous individual, 3 of the 4 possible allele combinations are inactive, predicting 75% loss of receptor function if all half receptor combinations are expressed equally at the cell surface. Other mechanisms may also contribute, including sequestration of downstream substrates by mutant heterodimeric receptors (15). The INSR also heterodimerises with the closely homologous insulin-like growth factor 1 receptor (IGF1R) (16), with IC INSR variants also causing loss of function of these hybrid receptors (12), though the clinical relevance of this remains speculative.

It is not yet clearly established that any extracellular (EC) INSR variant has dominant negative activity, and nor whether simple loss of expression of one allele has deleterious consequences (i.e. haploinsufficiency). Scattered case reports and case series have supported one or both of these possibilities (e.g. (17–24)), including a recent larger case series from the French National Referral Centre for Severe IR (25). Interest in *INSR* haploinsufficiency has further been piqued by the finding in UK Biobank that heterozygous *INSR* loss-of-function variants are associated with a benign lipid profile and adipose topography, although only nominally with diabetes (26).

The phenotypic spectrum associated with heterozygous *INSR* defects is narrower than that observed in individuals with biallelic *INSR* variants. Affected individuals most commonly present during puberty or early adulthood with diabetes, and/or clinical manifestations of IR including acanthosis nigricans (darkening and thickening of skin), or, in females, clinical features of excess ovarian androgen production (27). Unlike “common” type 2 diabetes, IR and PMOS (formerly PCOS(28)), often associated with obesity, metabolic dyslipidaemia (high serum triglyceride and low HDL-cholesterol) is usually absent in INSR-related IR and PMOS (29). In females the syndrome is often referred to as Type A IR (30), with no settled nomenclature for males. Another presentation increasingly described has been with symptoms of hyperinsulinaemic hypoglycaemia (31–39).

In this report we seek to clarify the phenotypic spectrum and natural history of IR caused by heterozygous *INSR* mutations, encompassing classical dominant negative mutations, and variants predicted to induce simple loss of function (LoF). The latter group of variants were ascertained by genetic testing after clinical presentations with dysregulated insulin action, on testing of parents of children with recessive receptoropathy, or were identified in genotyped cohorts with associated metabolic data.

## METHODS

### Ascertainment, ethics

PubMed was searched with terms including “monogenic insulin resistance”, “Type A insulin resistance”, “insulin receptor mutation”, and “INSR”. Case reports, series, trials and relevant reviews were selected, with secondary review of cited reports not captured by the primary search. In a subset of cases, up to date clinical information was sought from managing clinicians. In addition to published cases we included individuals with predicted LoF monoallelic *INSR* variants identified through genetic testing in large referral centres for IR in Cambridge (UK), Paris (France), the National Institutes of Health (Bethesda, USA), or as part of genetic testing for early onset diabetes and/or hyperinsulinaemic hypoglycaemia in Exeter (UK) or Chennai (India). We also included additional variants curated in ClinVar or the Human Gene Mutation Database (HGMD) for which a citation and clinical data were accessible. Finally, the metabolic profile of volunteers harbouring heterozygous known or likely LoF *INSR* variants in the Fenland prospective cohort study, based in the East of England (40), were assessed in the context of wider cohort data. For all cases, whether ascertainment was through clinical presentation with symptoms suggestive of a disorder of insulin action (= “probands”), or on screening of potentially affected relatives (= “relatives”), was noted.

### Data curation/cleaning

Amino acid numbering was aligned to the *INSR* MANE transcript NM_000208.4, corresponding to the longer B isoform with exon 11. Population allele frequencies were determined from gnomAD v4.1.1 (41) (accessed April 2026). For nonsense or frameshift variants the probability of nonsense-mediated decay (NMD) was determined using NMDetector (42); essential splice site variants were assessed using SpliceAI (43). Variants were classified as high-confidence complete loss-of-function (LoF) variants if they met any of: nonsense or frameshift variants predicted to be subject to NMD OR lying in the EC domain; essential splice site variants without predicted accessory splice site unmasking. (3) Deletion of ≥ 1 EC exon predicted to cause a frameshift. EC missense variants were additionally annotated with results from a recently reported multiplexed assay of variant effects (44).

The year of publication or of last clinical assessment (whichever was later) was used to group reports into three eras, demarcated by therapy-licensing landmarks: (A) before 1999 (pre thiazolidinediones); (B) 2000–2012 inclusive, (DPP-4 and GLP-1 agonist era, up to first SGLT2 inhibitor in 2013); (C) 2013 to present (rapidly advancing SGLT2 inhibitor and incretin therapy use).

Birthweight Z scores were computed using INTERGROWTH-21st (45) and BMI standard deviation scores (SDS) using CDC reference data (46), neither adjusted for ethnicity. Parents of origin and data on pregnancies of affected women (explicit or inferred from family structures) were documented. Organ- or system-specific clinical features, and treatments, were recorded dichotomously as present or absent, with accompanying free text. Longitudinal biochemical measures were recorded as single values (unique values reported or the highest in a series as indicated). Cohort frequencies are expressed as a range. The denominator includes either cases where presence/absence was specifically mentioned, or the whole group (assuming the feature was/was not present if not mentioned), giving very conservative upper and lower bounds. The full dataset is in **Additional File 1**; categorical analysis is in **Additional File 2**.

### Population Reference Data

Fasting insulin and adiponectin concentrations for adults were contextualised against the Fenland study (around 5,300 non diabetic women and 4,500 non-diabetic men, largely white adults from East England) (40, 47). Precise numbers varied by assay and are indicated. Whole-exome sequence data for the Fenland cohort were assessed using VerifyBamID (v2.0), requiring FREEMIX ≤0.05, and variants annotated using the Ensembl Variant Effect Predictor v114 (48) supplemented with REVEL, CADD, AlphaMissense, and LOFTEE plugins. For children <12 years old, IDEFICS age- and sex-specific reference centiles (3–10.9 years, eight European countries) (49) were used. For children >12 years old, NHANES 2011–2018 age-specific centiles (12–18 years, fasting glucose <5.6 mmol/L) (50), were used. Puberty-specific 97.5th centile thresholds from non-obese healthy subjects aged 5–80 years (51) were also used; pubertal status was approximated by age (<10 = pre-pubertal, 10–15 years = pubertal, >15 = post-pubertal), consistent with population-level median timing. For post-challenge insulin, a 2h insulin >986 pmol/L, (the maximum 2h post-OGTT insulin in healthy lean children 7.7–18.8 years) (52) was used as a hyperinsulinaemia threshold.

### Functional study of INSR p.M1180K

cDNA encoding the of human INSR B isoform (ENST00000302850) with a C-terminal myc tag was generated, and point mutations introduced with QuikChange XL (Stratagene), before verification by Sanger sequencing. Stable Flp-In CHO lines were generated by co-transfection with pOG44 and selection in hygromycin B, as previously described (53). Lysates (25 μg protein, DC assay) were resolved on 4–12% bis-tris NuPAGE gels (MOPS), transferred to nitrocellulose, and probed with antibodies against INSR β-subunit (sc-711) or β-actin (4967), followed by HRP-conjugated secondary antibody and ECL detection, as described previously (53). Cell-surface INSR expression was assessed by flow cytometry (BD FACSCalibur) on trypsinised cells labelled with 83–14 anti-INSR or mouse IgG, detected with FITC-conjugated anti-mouse IgG, with 25,000 events acquired per sample, as previously described (53). Serum-starved cells were stimulated with insulin for 10 min at 37°C. Myc-tagged receptor autophosphorylation was quantified using europium-labelled anti-phosphotyrosine (PY20) capture ELISA with time-resolved fluorescence (Tecan), as previously described (53).

#### Statistical Analyses

In analyses of variant distribution, individuals were the counting unit, with variants present in more than one individual counted separately. For analyses of IC/EC distribution and mutation type frequencies within the IR group, the denominator was all 240 probands; for gnomAD comparisons, the denominator was restricted to probands with missense or high-confidence complete LoF variants (n=222). A CADD PHRED ≥16 threshold was applied to gnomAD variants, as all proband variants with CADD scores had CADD ≥ 16. Large indels and whole-exon deletions, which cannot be CADD-scored, were classified directly as LoF. IC (407 aa) vs EC (975 aa) variant density ratios were compared using a two-sample Poisson rate ratio test (conditional exact binomial, Clopper-Pearson 95% confidence interval), back-transformed to variant density ratio (DR) scale. DR>1 indicates a higher per-amino-acid in the IC region.

Variant-type proportions and IC:EC ratio-of-ratios between cohort and gnomAD were compared using Fisher's exact test. Bonferroni correction was applied across missense and LoF rows; sensitivity analysis was performed across CADD thresholds 15–30.

To generate centile curves for insulin as a function of BMI or BMI-SDS, NHANES 2011–2018 (three two-year cycles; raw XPT data from https://wwwn.cdc.gov/nchs/nhanes/) and Fenland data were used. The 5th, 50th, and 95th centile curves were generated by quantile regression on log-transformed insulin, then back-transformed for display. Reference centile curves for fasting insulin vs age in pre-pubertal children were taken directly from IDEFICS (49); 97.5th centile thresholds for fasting insulin by pubertal stage (inferred from age) were from Hammel *et al* (51).

Categorical variables are reported as N (%) and continuous variables as median [25th, 75th]. Continuous between-group comparisons used Mann-Whitney or Welch's t test; birthweight SDS by parent of origin used Welch's t test. The one-sample Wilcoxon test assessed whether observed SDS differed from 0. Proportions were compared with Fisher's exact test. Relationships between fasting insulin and BMI were analysed by simple linear regression. Statistical analyses were performed using GraphPad Prism (GraphPad Software, Boston, MA, USA) or python.

#### Large Language Model Use

Large Language Models were used only for verifying internal consistency of data presented, and to aid text editing to reduce word count.

## RESULTS

### Case ascertainment

Rates of both case publication and clinical diagnosis of unpublished cases have increased markedly since roll-out of next-generation sequencing in clinical practice (Figure 1A), with numerous recent reports from regions historically underserved by genetic testing. 505 people with monoallelic rare *INSR* variants were identified (Figure 1B), including 281 from published reports (rare variants reported as causal for severe IR), and others from genetic testing centres with an interest in severe IR. Countries represented were UK and Ireland (198 individuals), Japan (75), France (57), US (49), China (26), Italy (23), Spain (11), Denmark (10), Turkey (9), with ≤3 each from other countries.

240 individuals (48%) were index cases, identified with confirmed or highly likely deleterious *INSR* variants as part of diagnostic testing after presenting with severe IR or hypoglycaemia. They were designated “probands”. All other individuals included were identified through testing of affected relatives of a proband with IR and at least one *INSR* variant, and were included based on carrying a familial heterozygous, predicted or proven deleterious *INSR* variant, irrespective of any evidence of clinically affected status. These 265 people were designated “relatives”.

### General Characteristics and Clinical Presentations of Individuals Studied

There was a marked female predominance (Male:Female = 1: 3.4) among probands that was strikingly attenuated among relatives diagnosed on family screening (M:F = 1: 1.3). Females moreover presented at a younger median age (13.0 [11.0–20.0] years) than males (19.8 [11.0–39.8] years, p=0.0013;) and were younger at last reporting age (20.0 [14.0–37.0] vs 34.3 [17.0–46.3] years, p=0.0002).

Among all probands, the most common reason for presentation was diabetes (46% of 214 probands with reason for presentation given), followed by clinical hyperandrogenism (43% of 161 pubertal females where a reason was reported), signs/symptoms of hyperinsulinemia (40%), and hypoglycaemia (11%) (**Additional File 3**). Male probands tended to present more often with diabetes (60 vs 42%) or hypoglycaemia (18 vs 9%), while female probands presented more often with hyperinsulinaemic manifestations (45 vs 26%); none of these sex differences reached significance. Probands with intracellular variants (“IC probands”) presented more often than those with extracellular variants (“EC probands”) with non-ovarian features of IR (45 vs 26%) but less often with diabetes (44 vs 53%) or hypoglycaemia (10 vs 14%), but again differences were not significant.

### INSR mutational spectrum

65% of the entire *INSR* cohort had purportedly pathogenic IC *INSR* variants, while the rest had EC variants (Figure 1B). Among the 240 probands, 75% carried IC variants and 25% EC variants. Missense variants accounted for 76% of all proband variants, high-confidence complete loss-of-function (LoF) variants for 16%, in-frame insertions/deletions for 3%, and large deletions for 4%. LoF variants were predominantly in the EC domain (32/39 = 82%), while missense variants were predominantly located in the IC domain (160/183 = 87%).

We compared the mutational spectrum in probands to that in gnomAD v4.1.1 (807,162 individuals (54)), restricting to missense and LoF variants — the two variant classes with distinct functional consequences well-represented in both datasets — with CADD PHRED scores ≥16, as 16 was the lowest score seen for any proband missense variant (Figure 2A). The proportion of missense variants was similar between probands and gnomAD (82% vs 87%; OR 0.7 [95% CI 0.5–1.0], p=0.04; **Additional File 1**). LoF variants were distributed across the protein in proportion to domain length in both groups (IC:EC LoF density ratio (DR) = 0.52 [0.20–1.21] for probands and 0.73 [0.51–1.03] for gnomAD, both NS, where DR = number of variants/number of EC or IC amino acids as appropriate). The *INSR* group showed strong enrichment for missense variants in the IC domain (DR 16.7 [10.7–27.0], p=3.92×10^−60^), far exceeding the modest enrichment in gnomAD (DR 1.2 [1.1–1.4], p=0.002; OR_adj 13.9 [8.8–22.8], p=1.1×10^−45^). This was robust across CADD thresholds from 15–30 (data not shown).

Nine of 12 unique IC nonsense or frameshift variants were predicted to lead to nonsense-mediated decay (NMD), suggesting that these may behave as heterozygous null variants (50% loss of function *in vivo*) rather than dominant negative expressed variants (i.e. >50% loss of INSR function *in vivo*). The single intracellular exon deletion and essential splice site variant in probands both affected the terminal exon. The remaining variants were small in frame deletions.

These observations are in keeping with current understanding that clinical expression of heterozygous *INSR* missense variants depends on dominant-negative activity of expressed intracellular variants, whereas LoF variants confer their effect largely independently of site within the receptor. IC variants clustered in key motifs: the ATP-binding loop, the catalytic loop (D1159, R1163, N1164) and the activation loop (Figure 2B). No pathogenic variants were identified in the three activation-loop tyrosines subject to trans-phosphorylation, but M1180 and R1201 — also in the activation loop — were the most commonly mutated residues. 25 probands had R1201 variants (15 R1201Q, 7 R1201W and 1 each of R1201G, R1201L, R1201P), and 22 had M1180 variants (19 M1180K, 2 M1180I, 1 M1180V), with an apparent founder effect in Ireland and the UK for M1180K. M1180 lies immediately downstream of the DFG motif, while R1201, 22 amino acids further along, makes a salt bridge with phosphorylated Y1190 to stabilise the active loop; mutations disrupting this salt bridge prevent adoption of the active conformation even after autophosphorylation. Other recurrent variants affected R1158 (15 R1158W, 3 R1158Q), P1205 (16 P1205L), H1157 (7 H1157R, 3 H1157Q) and G1146 (8 G1146R). Conversely, 90 variants (75% of those in severely insulin resistant probands) were unique, accounting for 38% of all probands.

Among the most frequently reported variants, R1201Q (55–58), R1201W (58), P1205L (56, 59), R1158Q (60) and G1146R (61) have previously been functionally characterised and shown to exhibit absent or severely reduced signalling activity. We now tested M1180K functionally, and showed that it too is normally processed and expressed at the cell surface (Figure 3A,B), but that it is unable to autophosphorylate in response to insulin stimulation (Figure 3C), as previously reported for M1180I (11, 62, 63).

### Clinical and Biochemical Expression of Heterozygous Extracellular INSR Variants

Unlike for expressed IC variants, no examples of dominant negativity have been biochemically demonstrated for any EC *INSR* variant to date. Moreover, whether the INSR exhibits haploinsufficiency – that is, whether there is a clinical/biochemical phenotype attributable to losing function of one *INSR* allele, is not a settled question. We thus set out to examine individuals with one pathogenic EC *INSR* variant in more detail.

We first assessed 6 reported multiplex families in which IR has been reported to co-segregate with a heterozygous EC *INSR* variant (19–23, 64), in five of which the *INSR* variant causes complete LoF. We computed two-point parametric LOD scores per family using a dominant model at the INSR locus, and combined LOD across families (**Additional File 4**). This analysis showed significant combined LOD score of >3.3 across a wide range of penetrances and rates of non-*INSR*-related phenocopy (i.e. non *INSR*-related IR), significantly bolstering the case that indeed heterozygous EC variants that abolish or severe impair INSR function are associated with IR.

Looking more widely at the population we describe, 25% of probands had EC variants, although under-reporting of heterozygous EC *INSR* variants, given current uncertainty about their pathogenicity, may have skewed this prevalence downwards. Forty-four percent of relatives had EC variants. We assessed the degree of IR seen in all those with heterozygous EC variants, whether probands or relatives, plotting available fasting and post-challenge plasma insulin concentrations against relevant normative data, distinguishing those from families with a monoallelic or a biallelic proband with the labels “M” and “B” respectively.

For adults, data from the Fenland study were used (40, 65). Among 35 adults with EC *INSR* variants, 30 (86%) showed fasting insulin concentrations greater than the 95^th^ centile of the Fenland population, when BMI was taken into consideration (Figure 4A,B).

For ages 12–18, NHANES served as comparator: all 11 with heterozygous EC *INSR* variants and available BMI had insulin concentrations above the NHANES 95^th^ centile (Figure 5A,B). For ages 3.25–10.9, IDEFICS data were used, with 9/13 above the 95^th^ centile (Figure 5C,D). Grouping by pubertal stage (inferred from age), 23/28 probands of pubertal age exceeded the stage-specific 95^th^ centile, but only 1/4 prepubertal children ≤8 years did (Figure 5E). 14/16 probands had post-challenge insulin meeting or exceeding a threshold derived from OGTT in obese children (Figure 5F).

The above analyses indicate that the large majority of probands with EC variants, at least of pubertal age onwards, exhibit severe IR, though studies of those presenting clinically with severe IR are subject to ascertainment bias. Study of relatives (“M” and “B” in Figures 4 and 5) indicate that they too exhibit marked IR, which may be smaller in magnitude than for probands. IR is seen in most adult relatives of both monoallelic probands and biallelic probands (Figure 4A,B). Far fewer data from relatives below 18 years precluded confident comment, though 3 of 4 relatives of monoallelic probands showed IR, but none of three relatives of biallelic probands.

In a complementary approach, we examined the exome-sequenced Fenland cohort for individuals with either EC LoF variants or EC variants previously reported in IR probands (Figure 4C–F). All 4 EC LoF variants showed fasting and/or post-challenge insulin above the 95th centile, while the single IC LoF variant (R1020*, predicted to cause NMD) was associated with insulin in the normal range. Heterozygosity for S350L (which causes Rabson-Mendenhall Syndrome when homozygous, e.g. (59–61)) showed normal fasting and high-normal post-challenge insulin; R256H, identified only in trans with an IC variant (unpublished, and still of indeterminate significance), showed a similar profile.

### Metabolic Features Across the Whole Population Studied

Among 403 individuals with reported diabetes status, diabetes was present in 56% (62% of females, 45% of males; p=0.0021), although diabetes was a commoner first presentation in males. This likely reflects absence of clinical hyperandrogenism as a competing sentinel manifestation in males. 48% with LoF variants had diabetes vs 58% with non-LoF variants (p=0.16). Diabetes prevalence was much higher in probands than relatives (72 vs 40%, p<0.0001), and comparable in probands with IC vs EC variants (72 vs 74%; p=0.8) (Figure 6A).

Fasting insulin was available for 298 patients (59%), with median [25^th^, 75^th^ centile] of 368 [155, 763] pmol/L (Figure 6C), an order of magnitude higher than in Fenland controls (men: 43 [17–114]; women: 35 [15–91]). Non-fasting or 2-hour post glucose tolerance test insulin concentrations (which we refer to as “post challenge”) were available in 178 patients (35%), with median [25th, 75th centile] of 3,267 [1,511, 6,000] pmol/L. Both fasting and post-challenge insulin concentrations were lower in EC vs IC (p<0.0001 and 0.0002, respectively) and lower in relatives than in probands (p<0.0001). Fasting insulin was slightly lower in those with LoF vs non-LoF variants, and post-challenge insulin trended lower (p-0.047 and 0.07, respectively) (Figure 6E). Fasting glucose showed no difference between IC vs EC, and LoF vs non-LoF, and was slightly higher in relatives than probands (Figure 6D), but challenge unmasked an increase in IC vs EC and probands vs relatives (Figure 6F). BMI SDS correlated positively with log10[fasting insulin] in individuals with EC (R^2^=0.09, p=0.046) but not IC (R^2^=0.005, p=0.7) variants (Figure 6G).

Hypoglycaemia was present in 55% of the 191 patients in whom hypoglycaemia status was reported. This included 69 probands with hypoglycaemia at any stage, 23 of whom had hypoglycaemia as a presenting feature, and 46 who were found to have hypoglycaemia after the time of initial presentation (Figure 6B). There were no differences between hypoglycaemic and non-hypoglycaemic groups in sex, or BMI SDS, nor in rates of diabetes. Patients with hypoglycaemia presented at a slightly younger median age compared to those without hypoglycaemia (13 [8,19] vs 14 [12,31] years, p=0.0031).

Interestingly, hypoglycaemia was almost as common among relatives as among probands (48 vs 59%, p=0.18, Figure 6B), and several examples of dominant familial patterns of hyperinsulinaemic hypoglycaemia were reported (e.g. (31, 39)). Hypoglycaemia, when present, occurred postprandially in 44% and on fasting in 11%, with no timing reported in many cases.

Patterns of hypoglycaemia varied across the lifespan varied. The median age at onset was 11 [0,16] years (range birth to 60). In 10 patients, hypoglycaemia was observed neonatally and resolved within the first weeks to months of life; some had an initial diagnosis of congenital hyperinsulinism (lowest glucose 1.1 mmol/L (31)). One patient presented first with hypoglycaemia during pregnancy (66); in others, hypoglycaemia was noted only after starting medications for diabetes (GLP-1 receptor agonist, glimepiride, or metformin); one patient improved on metformin; one developed hypoglycaemia only after bariatric surgery (25). Seven patients had asymptomatic hypoglycaemia detected on laboratory testing, most often post-OGTT.

Pancreatic imaging was reported in two patients: one had focally increased ^18^F-DOPA uptake suspicious for focal nesidioblastosis; the other showed no abnormalities on angio-MRI or ^18^F-DOPA PET/CT (34). Importantly, no differences in INSR mutation type, receptor region (EC vs IC), complete LoF status, or CADD score were seen between hypoglycaemic and non-hypoglycaemic patients, and many classical IC missense variants known to impair signalling severely were seen in hypoglycaemic patients, including M1180K (functionally characterised in this study) and R1201Q, reported previously in severe infantile hyperinsulinaemic hypoglycaemia (31)). Only limited functional study of a single EC variant has suggested gain of function for any INSR variant associated with hypoglycaemia (37).

Serum lipid and adiponectin concentrations were not systematically documented, and related phenotypes were often commented on only qualitatively in published studies. MASLD (any imaging or histological evidence of increased liver fat) was present in fewer than 15% of people in most groups despite severe IR, with a trend to greater prevalence in those with EC or LoF variants (Figure 7A). Eight out 9 patients in whom MASLD was noted had an EC variant; 3 also had hypertriglyceridemia. A similar pattern was seen for metabolic dyslipidaemia (elevated triglyceride and/or low HDL cholesterol; Figure 7B). Triglyceride concentrations were available in 38 patients (7.4%), and HDL-cholesterol in 27 (5.3%); median triglyceride was 0.8 [0.6,1.2] mmol/L and HDL-C 1.5 [1.1,1.8] mmol/L (Figure 7C,D), consistent with our previous observations of normal lipid profiles in INSR-related severe IR (29, 67). Hypertriglyceridemia (>1.7 mmol/L) affected 8 patients (21%), with BMI SDS comparable to non-affected. Only one patient (with a dominant-negative IC variant) had unexplained marked and persistent hypertriglyceridemia (peak 10.4 mmol/L) (68). HDL-C was low (<1.03 mmol/L) in 4 patients (15%). Adiponectin concentration was available for 39 individuals (7.6%). Median [25th, 75th] concentrations were 5.5 [3.0,8.5] mg/L in males (Figure 7E) and 10.0 [5.2,15.9] in females (Figure 7F), closely similar to non-diabetic people in the Fenland study (men: 4.8 [2.3–9.7]; women: 8.1 [3.6–16.3]). This is consistent with adiponectin concentrations being preserved or increased in *INSR*-related IR, in contrast with lipodystrophy or obesity-related IR (69–72). Adiponectin did not differ between probands and relatives or by IC/EC or LoF status.

### Growth

Birthweight SDS, adjusted for sex and gestational age, was available for 70 patients (14%). Median [25th, 75th] was −1.0 [–1.8,–0.2]; p<0.0001 vs general population (Figure 8A). No subgroup differences were seen for IC/EC, LoF/non-LoF, or proband/relative; those with affected mothers tended to have higher birthweight than those with affected fathers (–1.4 [–1.8,–0.9] vs −0.5 [–1.6,0.1], p=0.09; Figure 8B).

BMI SDS was available in 246 individuals (49%). Median [25^th^, 75^th^ centile] BMI SDS was 0.7 [−0.2,1.4] (p<0.0001 for general population comparison). BMI SDS was lower in individuals with IC variants compared to those with EC variants (p<0.0001) with no differences between probands vs relatives or those with LoF vs non-LoF variants. Median BMI SDS was above 0 for all subgroups (p<0.0001) (Figure 8C).

Only 12 individuals (2.3%) had short stature noted. Comments about body fat distribution were made in 24 individuals, of whom 17 were described as having relatively higher body fat in the trunk and/or abdomen (i.e. android body shape), while 7 were described as having relatively higher body fat in the lower extremities (i.e. gynoid body shape).

### Ovarian Manifestations

Since the original Type A IR description (30), ovarian hyperandrogenism, ovulatory dysfunction, and ovarian enlargement have been recognised as common features of INSR-related IR in women, attributable to the co-gonadotrophin activity of hyperinsulinemia (73), which persists despite INSR dysfunction (27). Consistent with this, hyperandrogenism and/or PMOS were reported in 82% of 222 postpubertal females where commented. PCOS prevalence was comparable in IC vs EC and LoF vs non-LoF, but much more common in probands vs relatives (90% vs 56%, p<0.0001; Figure 8D).

IR and compensatory hyperinsulinemia have also been associated with early puberty and/or accelerated pubertal tempo, via co-gonadotrophin and central HPG-axis effects (74). This was less apparent here: among 30 pubertal female patients with reported menarchal age, median (25th, 75th) was 13.5 (11.3, 16.0) years. Two patients had thelarche before age 8; 11 had primary amenorrhea despite otherwise advanced puberty.

All but 2 neoplasms reported were ovarian (Figure 8E), including a steroid-secreting ovarian tumour in a 33-year-old woman with R813* (unpublished). An ovarian dermoid cyst, serous cystadenoma, and serous papillary cystadenofibroma occurred in females aged 8, 17, and 14. Beyond the ovaries, two endometrial cancers (M1180I, age 36, unpub.; W1202*, age 27 (75)) and two breast cancers (age 49; and the mother of an 8-year-old proband, maternal age unreported (76)) were reported.

### Fertility and Pregnancy

Among the women studied, 80 had directly documented pregnancies (51 published), with a further 19 inferred from familial relationships, accounting for 121 documented pregnancies and a minimum of 140 including inferred (Figure 8F). The mean number of pregnancies per woman (for 55 women with documented count) was 2.2 (median 2, range 1–8). For 30 women, substantive comments encompassed 69 pregnancies: live births were confirmed or assumed in 56 (81%), and adverse perinatal outcomes in 11 (16%: 5 neonatal or early post-neonatal deaths from 4 pregnancies, 3 stillbirths or foetal losses, and 4 miscarriages), and 2 terminations. The apparently low miscarriage rate (5.8%, vs ~15% background (77)) almost certainly reflects under-ascertainment of early losses in reports focused on metabolic phenotyping. Stillbirth and perinatal death rates appear elevated compared to high-income country backgrounds (78). Preterm birth occurred in 13 pregnancies (19%), higher than the global 10% estimate (79). Assisted conception was required in at least 4 pregnancies across 3 women.

Gestational diabetes (GDM) was explicitly documented in 16 pregnancies across 13 women, affecting 16% of the 80 women with directly documented pregnancies (13% if 19 inferred pregnancies are included) and 43% of the 30 women with substantive pregnancy comments. Even the lower estimate of 13% exceeds the European background rate of 5–8% (IADPSG criteria (80)), but aligns with the global IADPSG rate of ~14% (81). Reports of high insulin requirements were scattered (e.g. up to 480 units daily in a woman with a heterozygous in-frame deletion of L1026); the earliest reported GDM onset at 9 weeks suggests pre-existing diabetes unmasked by pregnancy. In one woman heterozygous for R1119W, GDM occurred in four of six pregnancies. Neonatal hypoglycaemia was documented in 4 pregnancies (6%), well below the ~50% rate for neonates with established risk factors (82).

Marked heterogeneity in pregnancy outcomes was reported even for women with the same INSR variant. For women heterozygous for R1201Q these ranged from uneventful pregnancies through severe IUGR associated with octreotide treatment for hyperinsulinaemic hypoglycaemia (leading to elective terminations), to 3 spontaneous miscarriages. In women heterozygous for M1180K outcomes included well-controlled GDM with healthy term infants and 5 pregnancies resulting in foetal or neonatal death, and/or extreme prematurity (83).

### Extraovarian soft tissue overgrowth

As in the ovary, high insulin retains the ability to drive growth of some soft tissues despite INSR dysfunction — most prominently skin. Acanthosis nigricans status was reported in 229 patients and was present in 79% of females and 67% of males (p=0.09); higher in probands with IC than EC variants (88 vs 66%, p=0.0037) and in probands vs relatives (83 vs 60%, p=0.0003) (Figure 8G). Pseudoacromegaly and related soft-tissue overgrowth, reported in idiopathic (e.g. (84–87)) and lipodystrophy-related (e.g. (88–90)) IR, were not noted. Although IR has been associated with increased solid cancer risk (91), the only extraovarian cancers were an HPV-associated in situ cervical carcinoma (age 20, unpub.) and a pancreatic cancer (man in his 60s, unpub.).

### Other Organ-specific Features

At the most severe end of the monogenic IR spectrum, caused by biallelic INSR variants, a variety of additional organ-specific features are well described, including but not limited to nephrocalcinosis, hypertrophic cardiomyopathy, hepatic cholestasis and sometimes coagulopathy (9). None of these were reported in the monoallelic cases summarised in this report. Biallelic INSR defects are also often reported to result in neurocognitive impairment and frequent, severe infections. While these features were reported in scattered cases in the current series, these appeared commensurate with the degree of dysglycaemia reported rather than being independent syndromic features.

### Treatment

Among 505 patients, 235 had documentation regarding medication use for diabetes, IR, or hypoglycaemia (Figure 9; **Additional File 1)**; for the other 270, absence may reflect either non-use or non-reporting. Among 235 documented, 191 (81%) used ≥1 medication for diabetes/IR, 33 (14%) used none, and 14 (6%) used medications for hyperinsulinaemic hypoglycaemia (diazoxide, octreotide analogues, glucagon). Of treated patients, 130 (68%) used a single drug and 57 (30%) used ≥2. Biguanides (metformin in all but one early phenformin case) were the commonest anti-diabetic class (62%), followed by insulin (34%); 2–6% used SGLT2 inhibitors, incretin agonists, sulfonylureas, DPP-IV inhibitors, rhIGF-1, thiazolidinediones, or α-glucosidase inhibitors. Unspecified oral hypoglycaemic agents were used by 20%. Patterns of medication use tracked patterns of diabetes drug licensing (Figure 9; **Additional File 1**): rhIGF-1 dominated before 2000, and biguanides between 2000–2012. Metformin has remained predominant (58%) since 2013, but with growing SGLT2-inhibitor (7%) and incretin agonist (5%) use. Insulin dosing was highly variable (total daily 4–400 units).

Quantitative or qualitative efficacy of individual drugs was not reported in most cases. Biguanides had the most robust qualitative evidence for efficacy, with improved glycemia noted in 25 cases, and lack of improvement only in 2 cases. Other examples of improving control were documented, but were difficult or impossible to interpret given concurrent interventions in many cases, lack of controls, and the underlying highly dynamic natural of history of IR across puberty.

## DISCUSSION

As early as 4 years after the first reports of *INSR* variants in severe human IR, the broad mutational spectrum, mechanisms of LoF, and associated syndromes were reasonably well delineated (8, 92), as reflected in recent syntheses (e.g. (9)). Important clinical and scientific questions remain unresolved, however, and the exponential increase in case ascertainment since NGS entered clinical practice now provides rich substrate for an update. Many findings adduce numbers to phenomena well recognised by those managing monogenic IR: peripubertal presentation, with females presenting earlier and more often as index cases, driven by ovarian manifestations and greater female severity. Early-onset diabetes is common in both sexes and the commonest first presentation in males.

Missense variants in people presenting with IR are approximately 14-fold enriched in the IC region compared to gnomAD, while LoF variants are distributed in proportion to IC and EC domain lengths. These observations align with current understanding that clinical expression of heterozygous *INSR* missense variants depends on dominant-negative activity of expressed intracellular variants, while LoF confer their effect largely independently of site. Lack of EC dominant-negative missense variants may reflect the multiple insulin-binding sites and conformations of the ligand-binding region (93), with asymmetric contributions of hal-freceptors to each binding site and a single insulin molecule sufficient to trigger signalling (94, 95); a single point mutation could therefore not readily compromise signalling by a wild-type/mutant heterodimer.

IC missense variants cluster within well-defined tyrosine-kinase motifs involved in substrate positioning and catalysis, with multiple recurrences notably in the catalytic loop and activation-loop termini. We confirm complete loss of receptor signalling for one of the most common variants (M1180K), although its global frequency is likely overestimated by a strong UK/Irish founder effect. No pathogenic variants have been reported in the three phosphorylatable tyrosines of the activation loop, likely reflecting that mutation of any single one only partially inhibits receptor function (96, 97).

An important contribution of this study is to evaluate whether heterozygous LoF EC variants cause or predispose to IR. Several reports have suggested this (e.g. (17–21, 25)), without settled consensus, and many diagnostic labs do not report such variants as disease-causing alone. Our statistical evaluation of the combined LOD score from these reported families lends weight to previous assertions of co-segregation. Furthermore we now show that the large majority of all such patients have IR — sometimes severe — at least from puberty onwards, with a particularly marked pubertal interaction; evidence in prepubertal children is sparse but IR may be much less severe. EC variants were associated with milder IR than IC, consistent with lack of dominant-negative activity. BMI was higher with EC variants, and adults showed modest evidence of an EC-specific obesity interaction — supporting the notion that IC-variant IR is more strongly genetically determined, while EC variants interact with stressors such as obesity and/or puberty. In murine models, while no IR was apparent in heterozygous *Insr* knockout animals, double heterozygous knockout of *Insr* and *Irs1*, which encodes a key downstream signal transducer, did synergise to produce IR (98). The effect of dietary or hormonal cofactors on insulin sensitivity has not been subjected to the same concerted analysis in mice, however.

Some EC variant carriers presented with clinical manifestations of IR, but others were relatives of probands with either monoallelic or biallelic variants. The finding that both these subgroups of relatives exhibit IR, at least in adults, strengthens the case for a causal role of heterozygous EC variants in IR, though some ascertainment bias may persist. Genetic ascertainment from the general Fenland population mitigates this further and confirms that heterozygous EC LoF variants associate with IR in nearly all cases. Although deleterious missense variants were not associated with IR in this population, the weight of evidence now favours the view that heterozygous loss of INSR function causes IR in most carriers, albeit with variable penetrance and expressivity.

Agnostic exome-wide gene burden studies have shown that *INSR* LoF variants are associated with metabolically healthy waist-hip ratio adjusted for BMI, and with lower serum triglyceride and HDL-cholesterol (26). This is consistent with our lipid data and suggests EC LoF variants, like dominant-negative IC variants (29), may produce a distinct IR sub-phenotype that decouples IR from “metabolic” dyslipidaemia. Whether this confers different long-term cardiometabolic risk than obesity-related IR remains unclear.

Hypoglycaemia is a recognised feature of biallelic and, less frequently, monoallelic INSR-related IR, and is often the presenting feature (31–39). Indeed, after reporting of a large pedigree with dominant hypoglycaemic presentations (31), OMIM assigned the distinct disease entity Hyperinsulinaemic Hypoglycaemia 5 (HH5; OMIM#609968) to *INSR*. We show that hypoglycaemia is far more common than often appreciated in monoallelic insulin receptoropathy, documented in more than half of those studied at some point, and presenting in 12% of probands in whom status was reported. Variants seen in people with the full range of hypoglycaemic presentations — from neonatal hyperinsulinaemic hypoglycaemia (e.g. (36, 99)) to late-life postprandial hypoglycaemia (95) — have been functionally shown to be LoF, including M1180K studied here (36). Only a single report offers limited evidence for gain-of-function (37). This indicates that biochemical gain of function of mutated receptors does not need to be invoked to explain hypoglycaemia.

Hypoglycaemia was a common reason for discontinuation of therapies not normally associated with hypoglycaemia, including metformin and GLP-1 agonists. Given the high frequency of spontaneous hypoglycaemia in treatment-naïve *INSR*-related IR, it is unclear whether these therapies truly unmask hypoglycaemia or if pre-existing hypoglycaemia is instead detected on increased monitoring after initiation — but caution and counselling about hypoglycaemia risk on commencing therapy are warranted.

Insulin is an important growth factor *in utero*, and babies with biallelic pathogenic *INSR* variants exhibit severe *in utero* growth restriction (9). This has not previously been analysed at scale for monoallelic pathogenic variants. We now show that both IC and EC variants lower birthweight, albeit mildly. This effect may be more marked when genotype of the affected foetus is mismatched with that of the mother, although this difference does not reach statistical significance. Conversely, however, monoallelic variants increase BMI SDS. Whether this is primarily driven by reduced height, increased weight, or both, or simply reflects the population shift toward obesity compared to normative data from the 1960s, remains unclear.

Well-established adverse effects of IR and hyperinsulinaemia on ovarian function (27) — hyperandrogenism, ovarian enlargement, arrested follicular development, oligomenorrhoea or primary amenorrhoea — are confirmed in this series. We also document several neoplasms, including a steroid-secreting ovarian tumour in a 33-year-old woman with an EC nonsense variant, consistent with neoplasms previously described in lipodystrophic severe IR (27). Two endometrial and two breast cancers were also reported, and both tumour types are epidemiologically associated with IR; increased unopposed oestrogen exposure from ovarian effects of hyperinsulinaemia is a plausible mechanism. IR may also have contributed to two benign ovarian neoplasms (serous cystadenoma and serous papillary cystadenofibroma).

Fertility and pregnancy outcomes are of particular importance for dominant severe IR presenting especially in young females. Prior literature addresses them only piecemeal, and although serial complicated pregnancies (e.g. (83, 100, 101)) have been described, the scarcity of reports itself implies that outcomes are generally good. Across at least 140 documented or inferred pregnancies we report a lower miscarriage rate than population references — almost certainly under-ascertainment, since early losses are rarely documented in metabolic-focused reports. Stillbirth, perinatal death and preterm birth were modestly increased, but broad historical/geographic spread and incomplete documentation make these low-confidence conclusions. The high GDM rate (13–43% by denominator) is more robust. High insulin requirements or other glycaemic challenges in pregnancy were only rarely described, perhaps reflecting the mitigating effect of non-INSR-dependent placental glucose clearance in pregnancy.

As we documented in a recent systematic review, the evidence base underpinning treatment of monogenic severe IR is weak, relying on uncontrolled case series and case reports with sparse documentation of response and likely reporting bias favouring positive outcomes (102). This report confirms that dearth of high quality evidence, but does document evolving trends in medication use across different eras of diabetes drug licensing. The frequency of hypoglycaemia at baseline and after initiation of therapies not generally associated with hypoglycaemia is of particular note, and may warrant ambulatory glycaemic assessment to establish baseline prior to prescribing.

This study draws on all available cases, published and unpublished, with proven or likely pathogenic monoallelic INSR variants. For some, only thin clinical details were available, with limited follow-up. Race and ethnicity were highly variably documented, precluding consideration of this dimension of variability, and biochemical assays were not standardised across centres and eras. Nevertheless, the study is of unprecedented scale in the field, lending weight to its findings.

## CONCLUSIONS

This largest synthesis to date of monoallelic *INSR*-related IR, spanning 505 individuals, supports several major conclusions. The mutational architecture confirms a clear mechanistic dichotomy: pathogenic missense variants are approximately 14-fold enriched in the intracellular tyrosine-kinase domain (consistent with dominant-negative activity), while LoF variants are distributed across the receptor in proportion to domain length (consistent with position-independent haploinsufficiency). We provide the strongest evidence yet that heterozygous loss of INSR function — including extracellular LoF and deleterious missense variants previously of uncertain significance — causes IR in most carriers, albeit with variable penetrance and a marked pubertal interaction. We suggest that diagnostic laboratories should accordingly consider reporting heterozygous EC INSR LoF or deleterious missense variants which convincingly lead to severe LoF, and intracellular LoF variants even if predicted to trigger nonsense-mediated decay. The decision to report should be guided by the reason for testing, with the strongest case currently for direct clinical manifestations of IR. When testing is triggered by diabetes, we suggest that further evidence should be sought for IR before clinical reporting.

Hypoglycaemia is a far more pervasive feature than generally appreciated in heterozygous insulin receptoropathy, documented in over half of individuals and underpinned by impaired or absent signalling (including M1180K, characterised here as normally expressed but signalling-dead). Clinicians should counsel patients on hypoglycaemia risk when initiating glucose-lowering treatment. Modestly reduced birthweight, increased BMI SDS, prevalent ovarian hyperandrogenism and excess gestational diabetes complete the phenotype; the treatment landscape continues to evolve on a weak evidence base.

## Supplementary Material

This is a list of supplementary files associated with this preprint. Click to download.

- BrownetalAdditionalFile121.8.xlsx
- BrownetalAdditionalFile421.8.docx
- BrownetalAdditionalFile31.9.docx
- BrownetalAdditionalFile221.8.docx

**Additional le 1: Master dataset of the monoallelic INSR cohort** (Excel workbook, .xlsx).

**Additional le 2: Categorical phenotypic features of patients across their observed clinical course** (.docx).

**Additional le 3: Frequencies of presenting features among probands** (.docx).

**Additional le 4: Collective LOD analysis of published families with dominant insulin resistance associated with heterozygous extracellular INSR variants** (.docx).

## Figures and Tables

**Figure 1.**
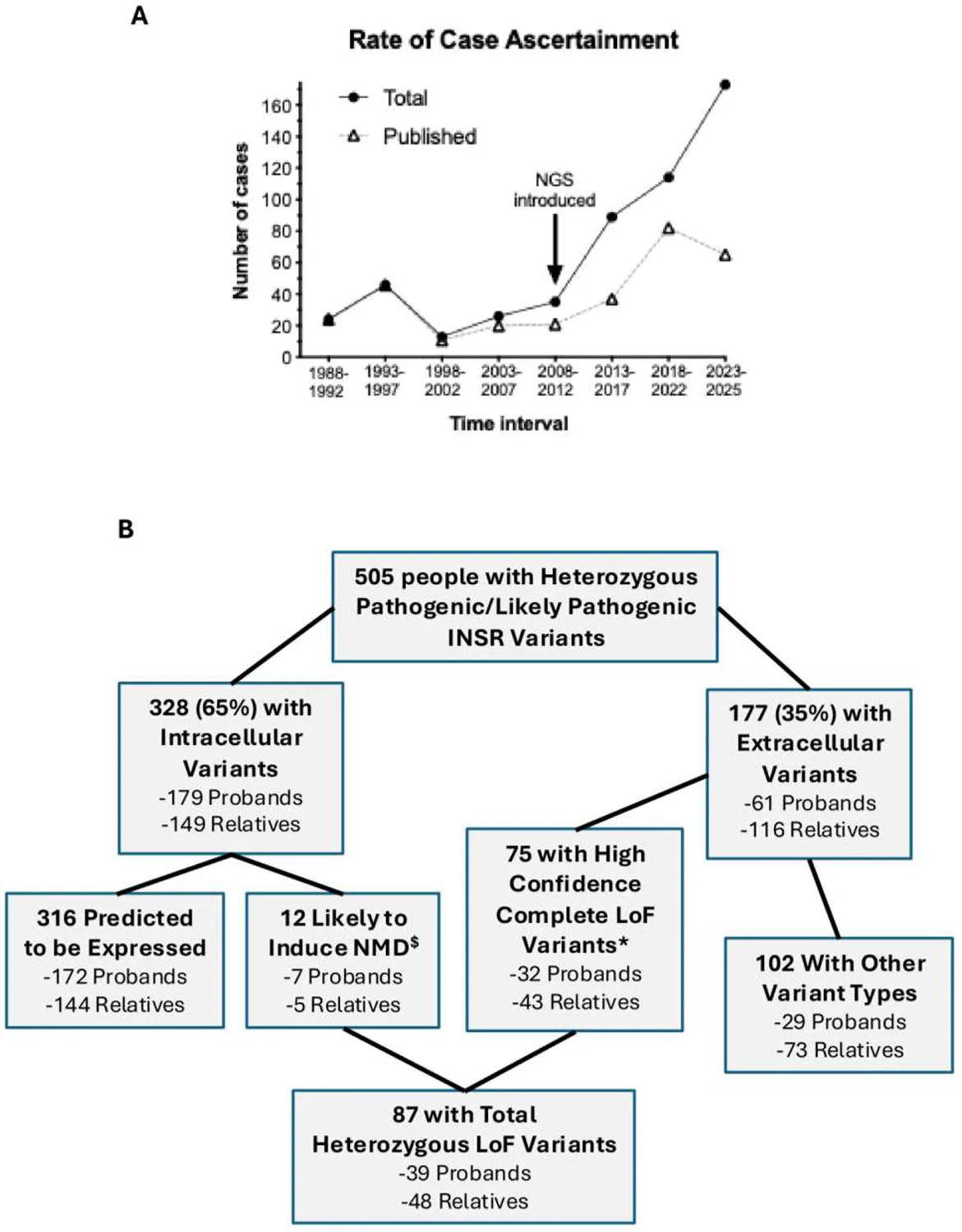
Ascertainment and Structure of INSR Group Studied. **A**. Historical trend in identification of published and unpublished cases, broken into the time windows indicated. NGS = Next Generation Sequencing. **B**. Substructure of the group of people studied with monoallelic INSR variants. monoallelic *LoF (Loss of Function) variants defined as nonsense or frameshift, or whole exon deletions if predicted to produce a frameshift or essential splice site variants if predicted to result in frameshift and not predicted to unmask cryptic splice sites by SpliceAI; ^$^ Nonsense Mediated Decay, as predicted by NMDetective.

**Figure 2.**
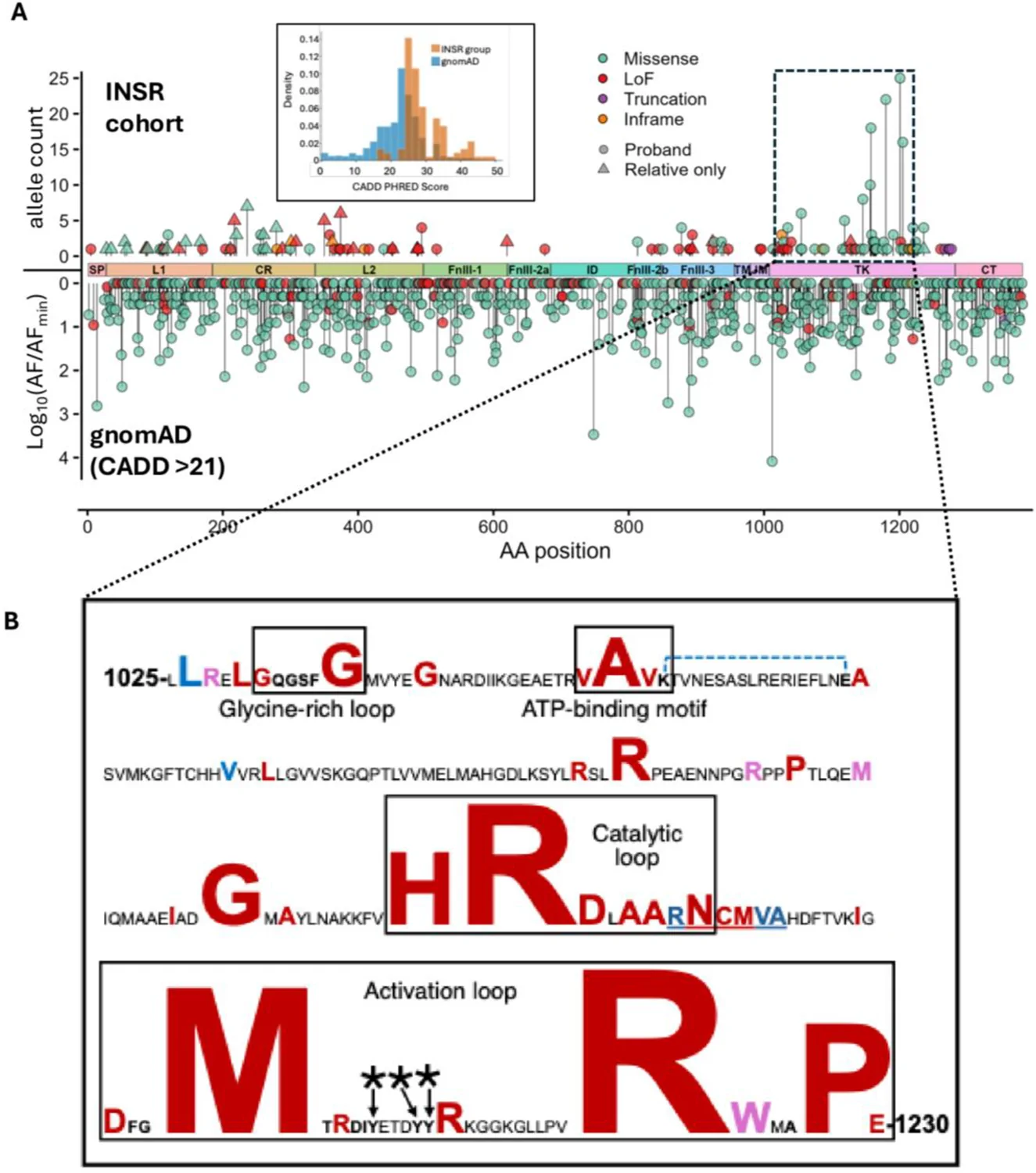
INSR Variant Distribution and Frequency in the Group Studied **A**. Distribution of *INSR* variants in probands and in the gnomAD dataset. CADD (Combined Annotation-Dependent Depletion) score distributions in probands and gnomAD are shown as an inset. Only variants with CADD scores greater ≥16 (met by 100% of proband missense variants) are shown in inset and lollipop plot for gnomAD, while only variant types also present in gnomAD are shown for probands. Allele counts are shown for probands, while for gnomAD the log of the ratio of the allele frequency (AF) for the variant concerned to the minimum AF in gnomAD (AF_min_) is shown. LoF = loss of function (nonsense or frameshift); **B**. Inset of the intracellular tyrosine kinase domain shows distribution of proband variants, with font size representing allele frequency. Key motifs are indicated in boxes. Asterisks mark the three activation loop tyrosine residues subject to autophosphorylation. The blue dashed line represents a key salt bridge. Red letters = missense variants. Blue letters = in frame deletions; Pink = LoF

**Figure 3.**
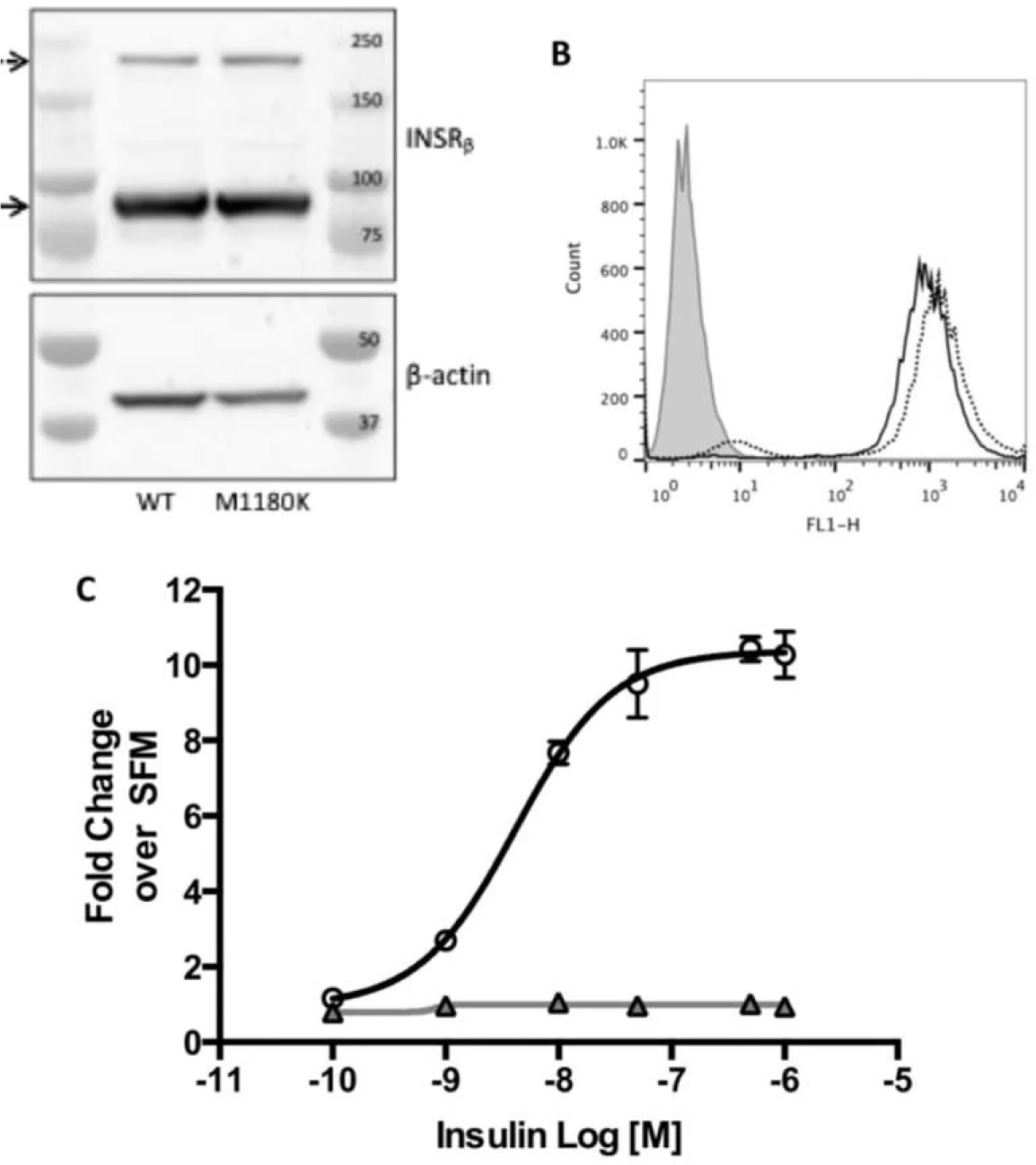
Functional Characterisation of the Common M1180K INSR variant. **A**. Immunoblot showing equal expression of total cellular mature INSR beta subunit (solid arrow) and proreceptor (dashed arrow) for wild-type and M1180K receptors **B**. Fluorescence Activated Cell Sorting trace showing equal cell surface expression of wild-type (dotted line) and M1180K (dotted line) receptors, detected using the human INSR-specific murine 83–14 monoclonal antibody and FITC-conjugated secondary anti-mouse IgG. The area under the trace obtained using a mouse IgG isotype control antibody on wild-type expressing cells is shaded grey. **C**. Insulin-induced tyrosine phosphorylation of wild-type (black line) or M1180K (grey line) insulin receptor. SFM = serum-free medium.

**Figure 4.**
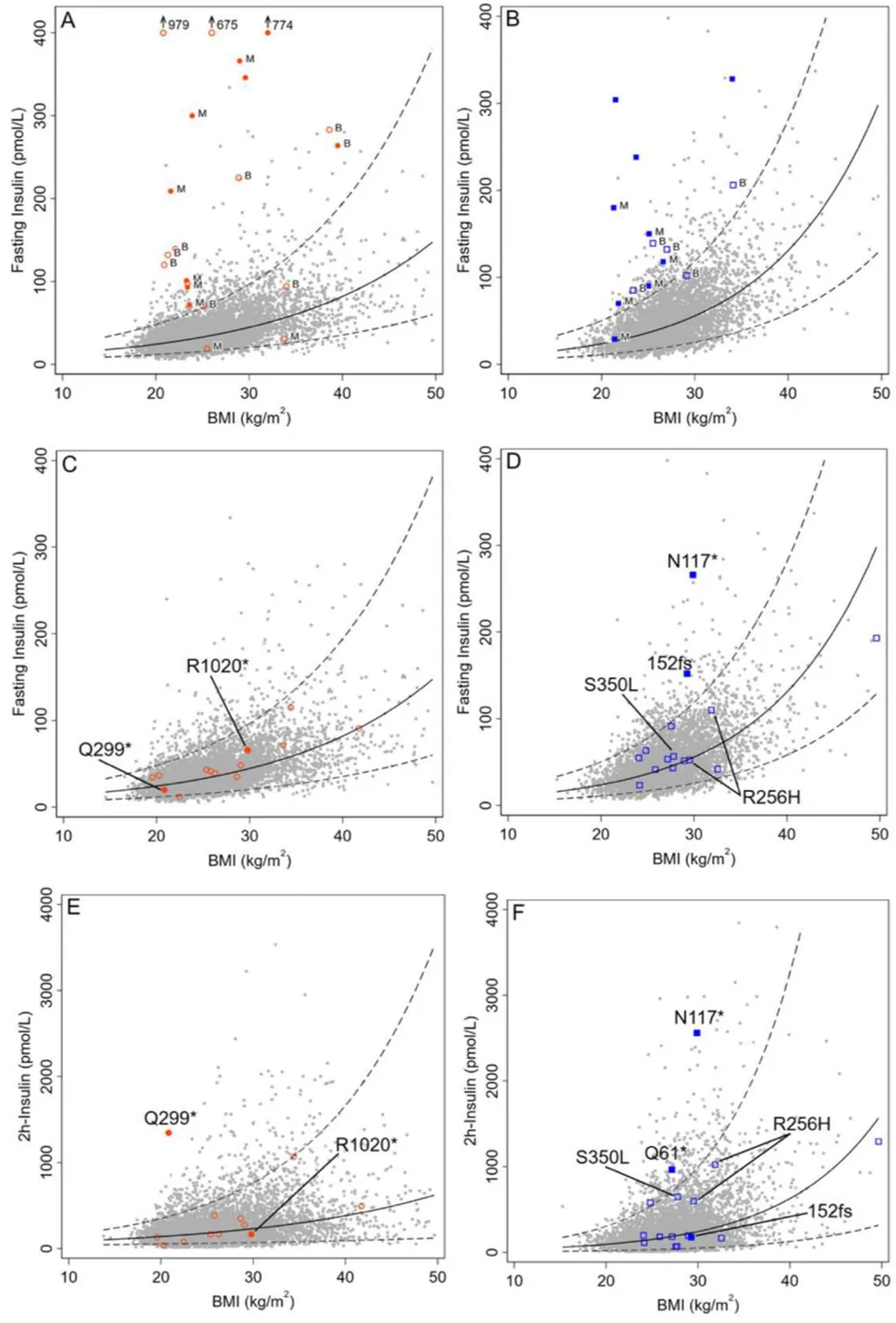
Fasting insulin concentrations of people with extracellular INSR variants referenced to age- and sex-matched controls Fasting plasma insulin concentrations and body mass indices (BMI) of **A**. females or **B**. males from the insulin resistance group who are over 18 years old with heterozygous extracellular INSR variants. Data are plotted with reference to 5,277 non-diabetic women (left) and 4,509 non-diabetic men (right) from the Fenland study. In both plots, data from females are plotted as orange icons and males as blue icons. People whose sex was not reported are plotted as black triangles, repeated on both male and female plots. Filled icons denote loss-of-function variants (nonsense, frameshift, essential splice site or whole exon deletions) and unfilled icons all other variants. Heterozygous relatives of monoallelic and biallelic probands are indicated by “M” and “B” labels respectively; **C-F**. The same Fenland data without the additional study cohort datapoints, this time labeling people with loss-of-function INSR variants (nonsense or frameshift), or missense variants previously identified in people with severe insulin resistance. **C** and **D**: Fasting plasma insulin for women and men respectively. **E** and **F**: Plasma insulin at 2 hours after a 75g oral glucose challenge for women and men respectively. In all plots the solid lines represent the smoothed 50^th^ centile and dashed lines the 5^th^ and 95^th^ centiles of **Fenland** data. Individual control population data points are shown in grey.

**Figure 5.**
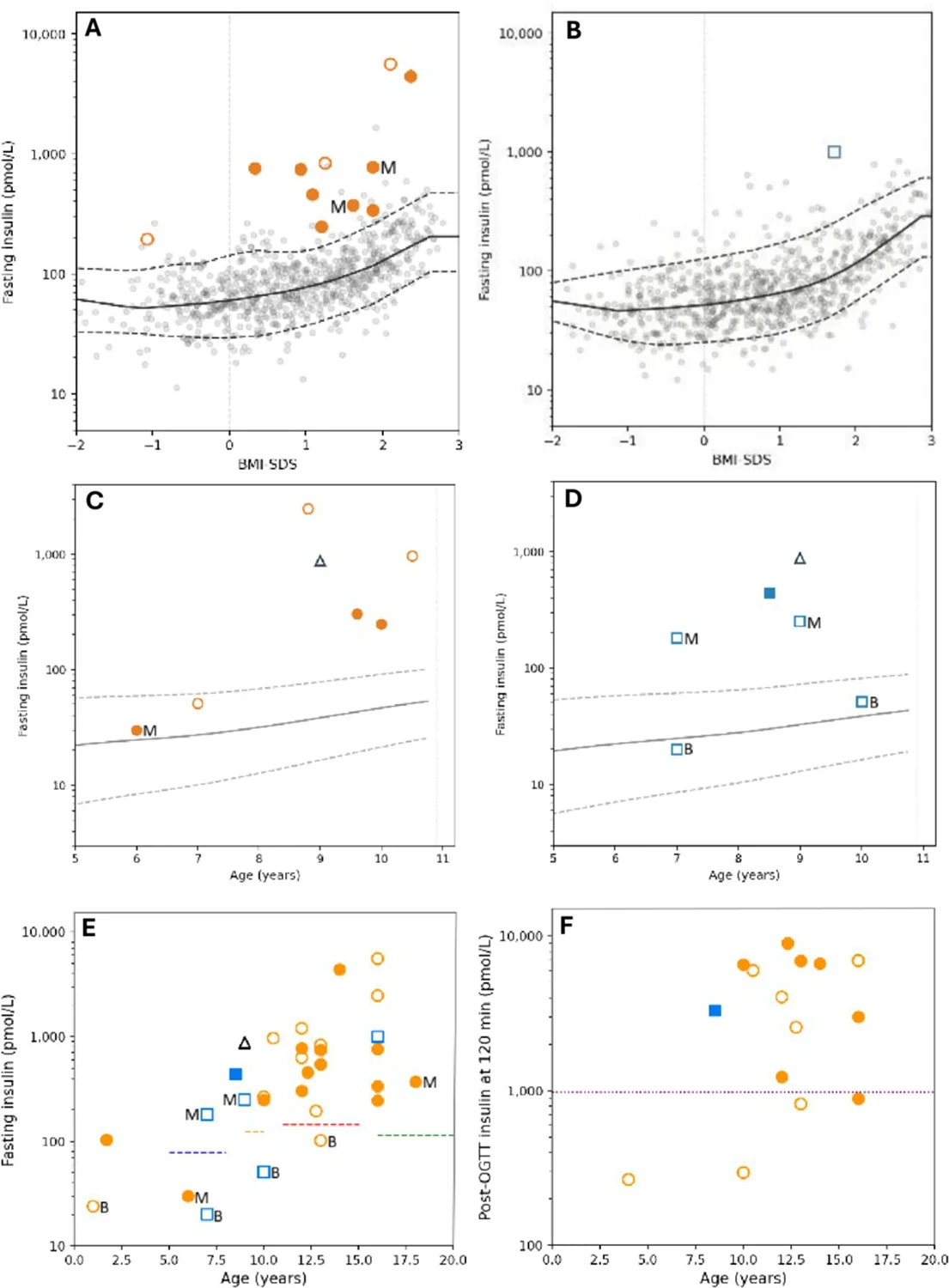
Insulin concentrations of people under 20 years old with extracellular INSR variants **A-B**. Fasting plasma insulin concentrations and BMI standard deviation scores (BMI-SDS) of **A**. Females and **B**. Males from 12 to 19 years old inclusive, plotted against data from 753 females and 754 males respectively of the same age from NHANES; **C-D**. Fasting plasma insulin concentrations and ages of **C**. Girls and **D**. Boys under 12 years old, plotted against published centiles from 3,434 girls and 3,640 boys respectively between 3.25 and 10.75 years old from IDEFICS. In **A**-**D** the solid lines represent the smoothed 50^th^ centile and dashed lines the 5^th^ and 95^th^ centiles of control population data. Individual control population data points are shown in grey in **A** and **B; E**. Fasting plasma insulin concentrations against age of people with heterozygous extracellular INSR variants. Dashed lines indicate 97.5^th^ centiles for different age ranges, drawn from Hammel et al (51), based on 461 people each from 5–8 and 9–10 years old, 929 people from 11–15 years old, and 331 people from 16–23 years old; F. Post challenge plasma insulin concentrations for people with heterozygous extracellular INSR variants. These were 75g oral glucose tolerance testing (OGTT) 2 hour values where possible. The dotted horizontal line at 986 pmol/L shows the maximum 2-hour post-OGTT insulin reported in 64 healthy lean children aged 7.7–18.8 years (52); In all plots, data from females are plotted as orange icons and males as blue icons. People whose sex was not reported are plotted as black triangles, repeated on both male and female plots. Filled icons denote loss-of-function variants (nonsense, frameshift, essential splice site or whole exon deletions) and unfilled icons all other variants. Heterozygous relatives of monoallelic and biallelic probands are indicated by “M” and “B” labels respectively.

**Figure 6.**
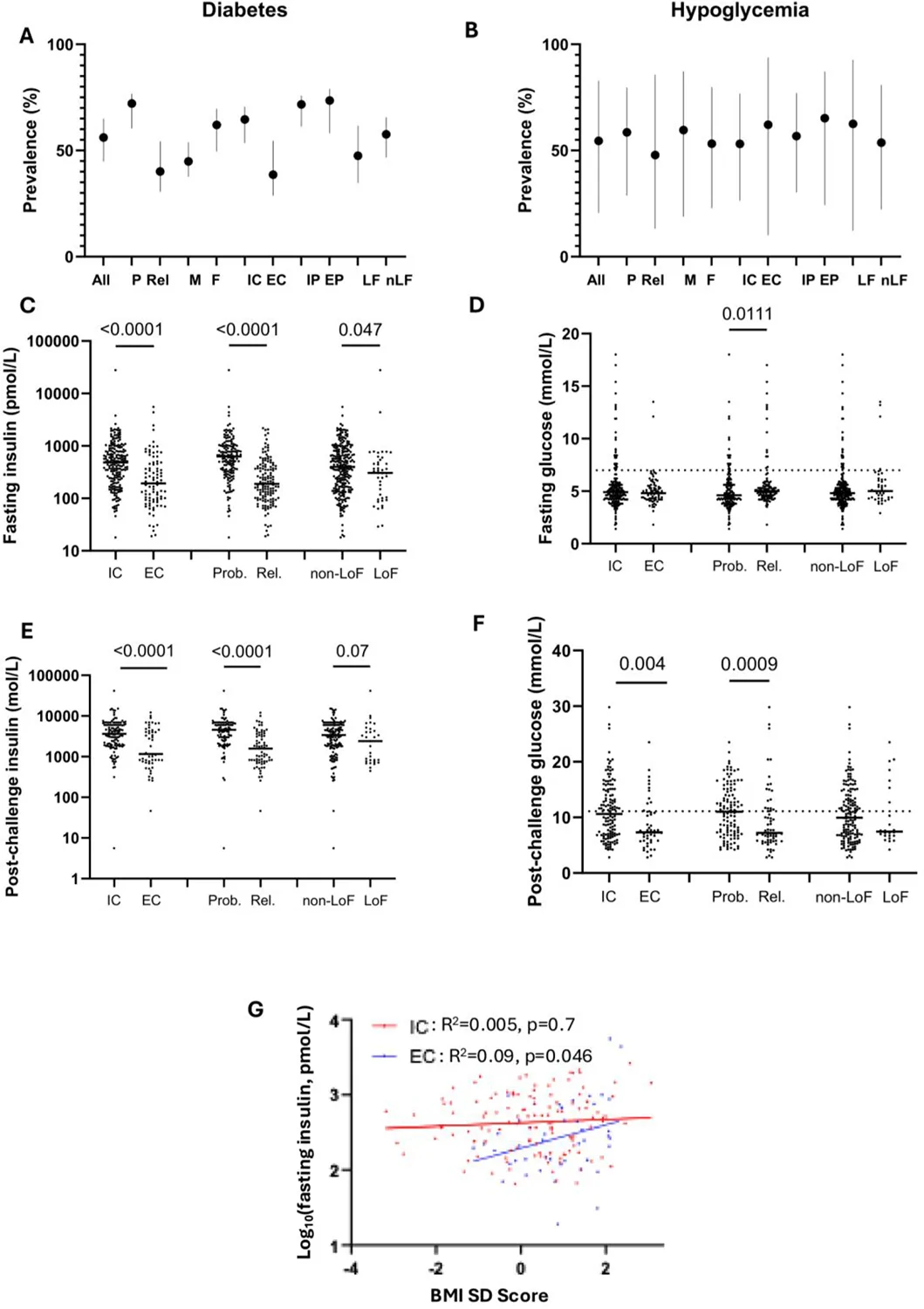
Glucose and Insulin Profiles and their Disorders in Monoallelic Insulin Receptoropathy Prevalence of **A**. Diabetes and **B**. Hypoglycaemia in the monoallelic INSR variant population. All = all people with heterozygous INSR variants; P = Probands; Rel = Relatives; M = Male; F = Female; IC = All people with IC variants; EC = All people with EC variants; IP = Probands only with IC variants; EP = Probands only with EC variants; LF = people with loss-of-function variants (frameshift, nonsense, or whole exon deletion/essential splice site without accessory splice sites); nLF = People with non loss-of-function variants (i.e. missense, in frame deletions, or splice variants with predicted alternative splice sites). In **D**-**G** circles represent point prevalence of all those where phenotype mentioned. Upper and lower bounds are conservative, defined by assuming that no mention of phenotype means presence (upper bound) or absence (lower bound). **C**. Log_10_(fasting plasma insulin concentration), **D**. Fasting blood glucose concentrations, with diabetes threshold marked as a dotted line, **E**. Log_10_(post challenge plasma insulin concentration), **F**. Post challenge blood glucose concentrations, again with diabetes threshold as a dotted line; Difference between paired datasets were tested by Mann-Whitney test and were non-significant where no p value is indicated **G**. Relationship between BMI SDS and Log10[Fasting plasma insulin concentration] for IC and EC groups.

**Figure 7.**
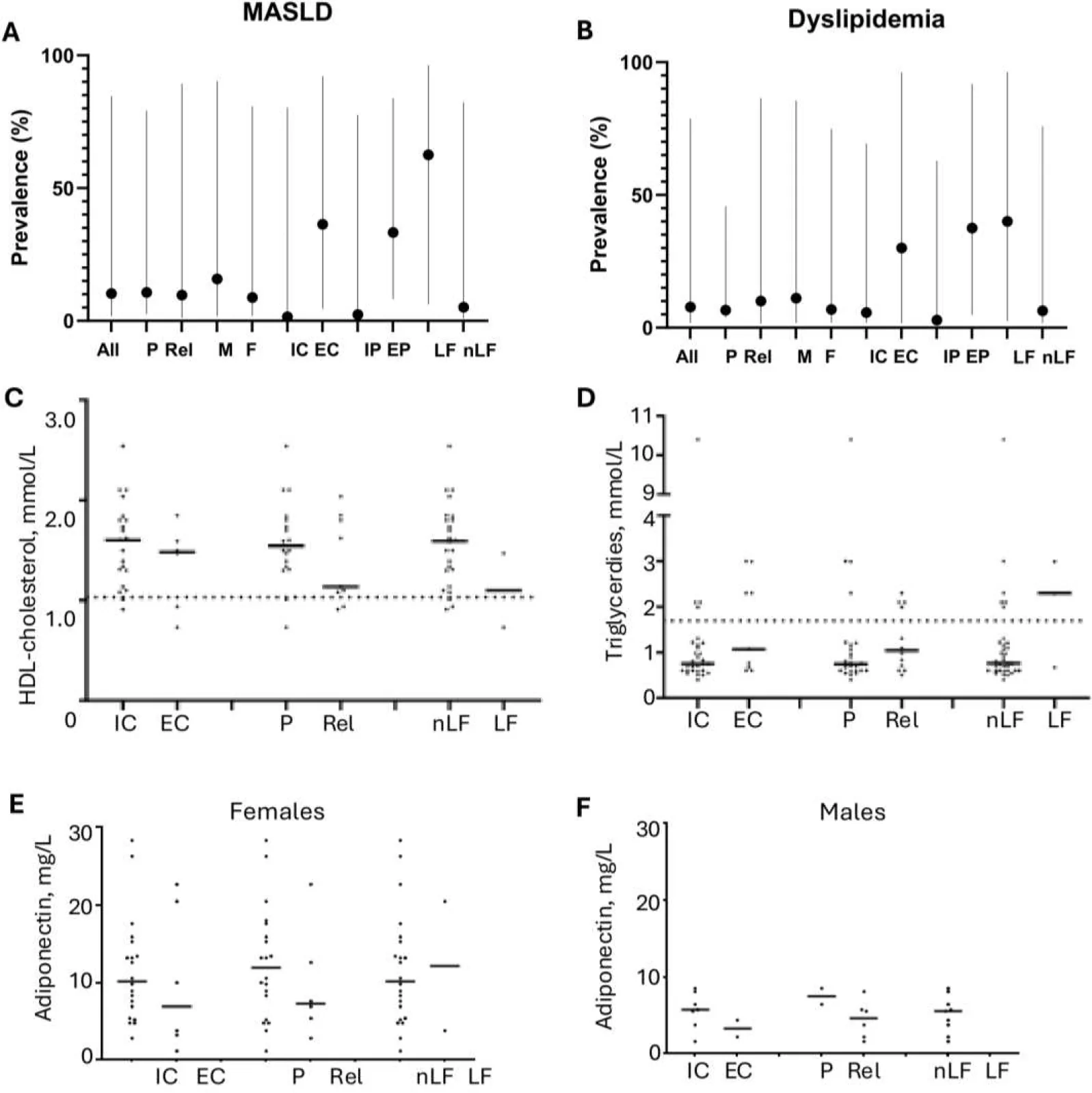
Wider Metabolic Features in People with Heterozygous INSR Variants Prevalence of **A**. Metabolic Dysfunction-Associated Liver Disease (MASLD); **B**. Dyslipidaemia (Defined as high serum triglyceride and/or low HDL-cholesterol). Circles represent point prevalence of all those where phenotype mentioned. Upper and lower bounds are conservative, defined by assuming that no mention of phenotype means presence (upper bound) or absence (lower bound); **C**. Serum HDL-cholesterol concentrations; **D**. Serum triglyceride concentrations; **E**. Serum adiponectin concentrations for the subset of females for whom data were available; **F**. Serum adiponectin concentrations for the subset of females for whom data were available. All = all people with heterozygous INSR variants; P = Probands; Rel = Relatives; M = Male; F = Female; IC = All people with IC variants; EC = All people with EC variants; IP = Probands only with IC variants; EP = Probands only with EC variants; LF = people with loss-of-function variants (frameshift, nonsense, or whole exon deletion/essential splice site without accessory splice sites); nLF = People with non loss-of-function variants (i.e. missense, in frame deletions, or splice variants with predicted alternative splice sites). Difference between paired datasets were tested by Mann-Whitney test and were non significant where no p value is indicated

**Figure 8.**
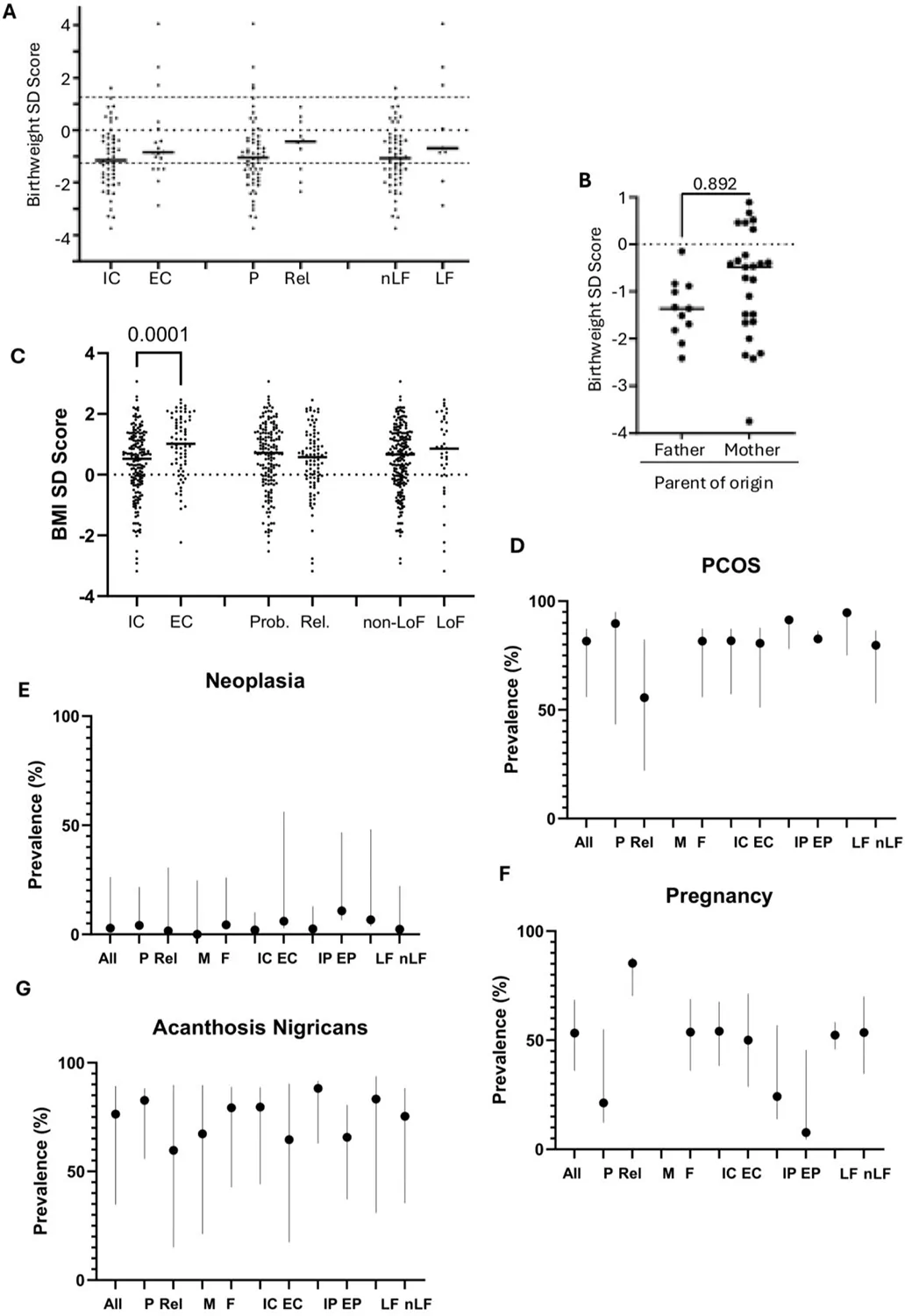
Growth and Reproductive Phenotypes in People with Monoallelic INSR Variants **A**. Birthweight Standard Deviation (SD) Scores for different groups of people with heterozygous INSR variants. All = all people with heterozygous INSR variants; P = Probands; Rel = Relatives; M = Male; F = Female; IC = All people with IC variants; EC = All people with EC variants; IP = Probands only with IC variants; EP = Probands only with EC variants; LF = people with loss-of-function variants (frameshift, nonsense, or whole exon deletion/essential splice site without accessory splice sites); nLF = People with non loss-of-function variants (i.e. missense, in frame deletions, or splice variants with predicted alternative splice sites). Dotted line, SDS of 0, indicating population mean. Dashed lines denote SD Scores of ±1.25, indicating cutpoints used to define small and large for gestational age. Values IC, P, and nLF groups were lower than the population median of 0 (p<0.0001, one sample Wilcoxon), while EC, Rel, and LoF groups were not different from 0; **B**. Birthweight SD Scores compared between those inheriting the INSR variant from father or mother. Welch’s T test was used to assess difference between groups; **C**. Body Mass Index (BMI) SD Scores; **D**-**G**: Prevalence of **D**. Polycystic Ovary Syndrome (PCOS), **E**. Neoplasia, **F**. Pregnancy and **G**. Acanthosis nigricans. In D-G circles represent point prevalence of all those where phenotype mentioned. Upper and lower bounds are conservative, defined by assuming that no mention of phenotype means presence (upper bound) or absence (lower bound). Only women of childbearing age are included in the pregnancy estimates.

**Figure 9.**
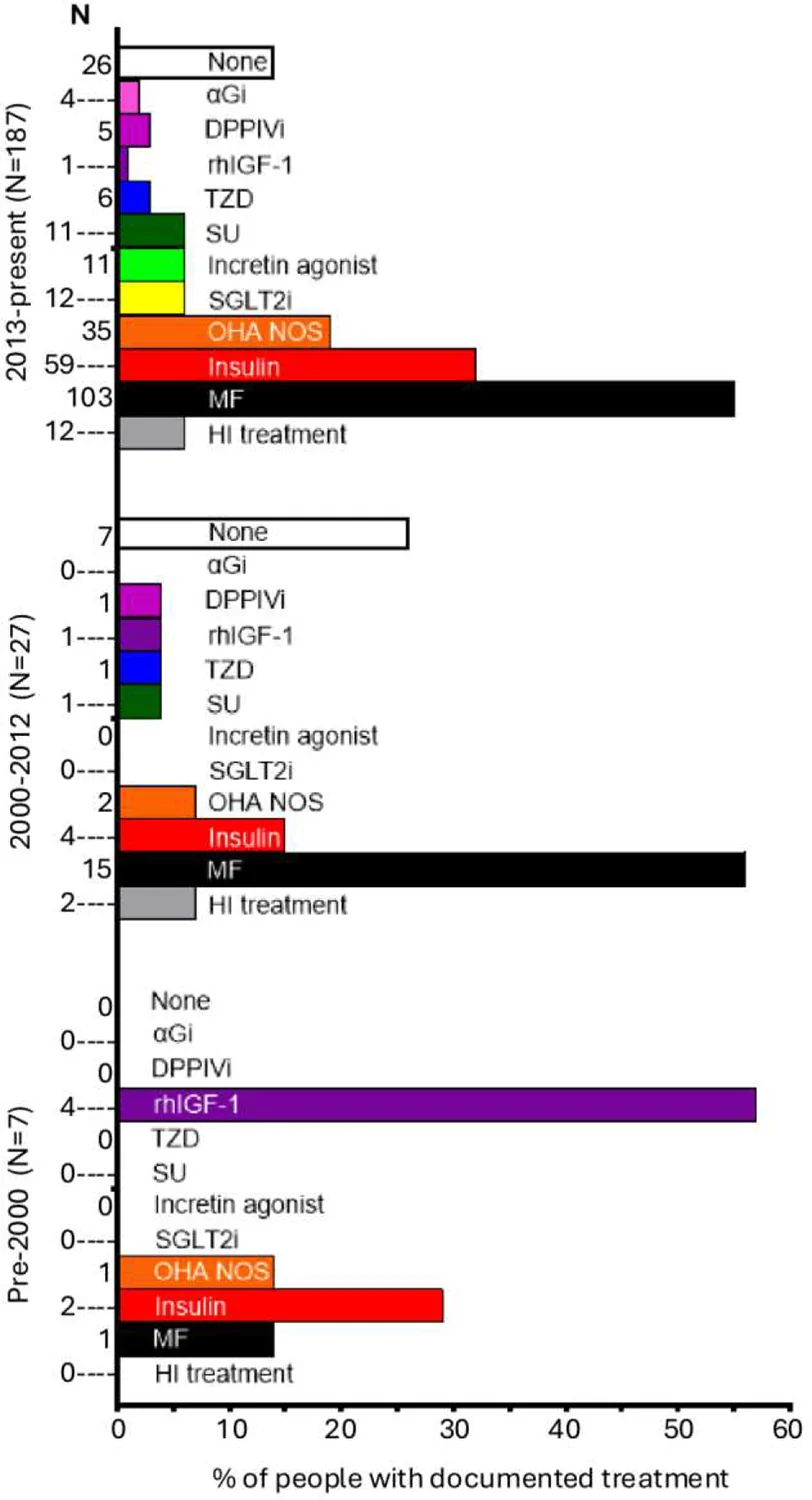
Summary of Changing Patterns of Drug Treatment for Monoallelic Insulin Receptoropathy with Time. Numbers of patients (N) treated with different glucose-directed therapies in each of three different time periods, namely Pre 2000, 2000–2012 and 2013-present. Total of numbers of patients from whom therapy was reported are shown. αGI = α-glucosidase inhibitor; DPPIVI = dipeptidyl-peptidase type IV inhibitor; rhIGF1 = recombinant human insulin-like growth factor 1; TZD = thiazolidinedione; SU = sulphonylurea; SGLT2i = sodium glucose transporter type 2 inhibitor; OHA NOS = oral hypoglycaemic agent not otherwise specified; MF = metformin; HI = hyperinsulinism

## Data Availability

All data are shown in the manuscript and additional files. Cell biology reagents and plasmids are available on reasonable request

## LIST OF ABBREVIATIONS

BMI: Body mass index
BSA: Bovine serum albumin
CADD: Combined Annotation Dependent Depletion
CDC: Centers for Disease Control and Prevention
CHO: Chinese hamster ovary (cell line)
DELFIA: Dissociation-enhanced lanthanide fluorescence immunoassay
DPPIV: Dipeptidyl peptidase IV
EC: Extracellular (domain)
EDTA: Ethylenediaminetetraacetic acid
EGTA: Ethylene glycol tetraacetic acid
F-DOPA: 6-[18F]fluoro-L-DOPA
FACS: Fluorescence-activated cell sorting
FCS: Foetal calf serum
GDM: Gestational diabetes mellitus
GLP-1: Glucagon-like peptide-1
gnomAD: Genome Aggregation Database
HDL-C: High-density lipoprotein cholesterol
HEPES: 4-(2-hydroxyethyl)-1-piperazineethanesulfonic acid
HPG: Hypothalamic-pituitary-gonadal (axis)
HPV: Human Papillomavirus
HRP: Horseradish peroxidase
IADPSG: International Association of Diabetes and Pregnancy Study Groups
IC: Intracellular (domain)
IDEFICS: Identification and Prevention of Dietary- and Lifestyle-Induced Health Effects in Children and Infants Study
IgG: Immunoglobulin G
IR: Insulin resistance
IUGR: Intrauterine growth restriction
LoF: Loss of function
MASLD: Metabolic dysfunction-associated steatotic liver disease
MOPS: 3-(N-morpholino)propanesulfonic acid
NGS: Next Generation Sequencing
NHANES: National Health and Nutrition Examination Survey
NMD: Nonsense-mediated (mRNA) decay
OR: Odds ratio
PBS: Phosphate-buffered saline
PCOS: Polycystic Ovary Syndrome
PMOS: Polyendocrine Metabolic Ovarian Syndrome
PCR: Polymerase chain reaction
PET/CT: Positron emission tomography/computed tomography
PMSF: Phenylmethylsulfonyl fluoride
RR: Rate ratio
SD(S): Standard Deviation (Score)
SGLT2: Sodium-glucose cotransporter 2
TBST: Tris-buffered saline with Tween-20
